# *Smyd1C* Mediates CD8 T Cell Death via Regulation of Bcl2-Mediated Restriction of outer Mitochondrial Membrane Integrity

**DOI:** 10.4172/2576-1471.1000163

**Published:** 2017-09-12

**Authors:** Hui Nie, Gary Rathbun, Haley Tucker

**Affiliations:** Department of Molecular Biosciences and the Institute for Cellular and Molecular Biology, the University of Texas at Austin, Austin TX 78712, USA

**Keywords:** Cell death, Apoptosis, Phosphorylation, *Smyd1*

## Abstract

The SET and *Mynd* domain 1 (*Smyd1*) locus encodes three tissue-restricted isoforms. Two previously characterized isoforms, *Smyd1A* and *Smyd1B***,** are heart and skeletal muscle-restricted histone methyl transferases. Here we report that a third, non-catalytic isoform, *Smyd1C***,** is expressed predominantly in activated CD8 T cells. While *Smyd1C* **-** deficient CD8 T cells undergo activation-induced apoptosis, neither of two classical mechanisms activation-induced cell death nor activated cell autonomous death are utilized. Instead, *Smyd1C* accumulates within both mitochondria and the immunological synapse where it associates with Bcl-2, FK506-Binding Protein 8/38 (FKBP38) and Calcineurin. This complex maintains Bcl-2 phosphorylation, enhanced mitochondrial localization, and restricted apoptosis of activated CD8 T cells. We suggest that CD8 T cell death is governed, in part, by *Smyd1C* regulation of Bcl2-mediated restriction of outer mitochondrial membrane integrity.

## Introduction

Apoptosis is critical to the fate of T cells. In response to T cell receptor (TCR) stimulation, T cells exhibit an exponential rate of expansion followed by a rapid decline and a return to a basal pool. Failure to induce apoptosis leads to elevated T cell levels and, often, to inflammatory or autoimmune disorders [1]. Reciprocally, aberrant elimination of T cells can lead to immunodeficiency. Therefore, a fine balance between cell proliferation and programmed cell death is necessary to achieve proper T cell homeostasis. This balance is governed by two distinct apoptotic pathways: activation-induced cell death (AICD) and activated T cell autonomous death (ACAD) [2]. While AICD is mediated through extrinsic pathways via the Fas death receptor or by signaling through TNF-α receptors, Bcl-2 family members play a central role in the removal of activated T cells by the ACAD mitochondrial intrinsic pathway [3–5]. During ACAD, a Bcl-2 family pro-apoptotic member, Bim, induces cell death [3]. Survival of the cell requires that anti-apoptotic Bcl-2 family members, including Bcl-2 and Bcl-XL, block Bim by interacting with Bim at the mitochondrial membrane [5]. Thus, the relative levels of mitochondrial anti-apoptotic versus pro-apoptotic members of the Bcl-2 family critically influence the regulation of apoptosis [2,6,7].

Bcl-2 is anchored to the mitochondria through its association with the mitochondrial chaperone, FKBP38 [6,8]. FKBP38 contains a peptidylprolyl cis-trans isomerase (PPIase) domain through which it binds the immunosuppressive drug, FK506. While its effect on apoptosis of cultured cells is controversial, knockout (KO) studies demonstrated that, *in vivo*, FKBP38 is required for inhibiting cell death in the developing neural tube. Bcl-2 family proteins are regulated by reversible phosphorylation modifications that control their activity and conformation. PKCα is the major Bcl-2 kinase [2,9], whereas dephosphorylation of Bcl-2 by calcineurin (CaN) enhances its anti-apoptotic activity [10,11].

**Figure 1:**
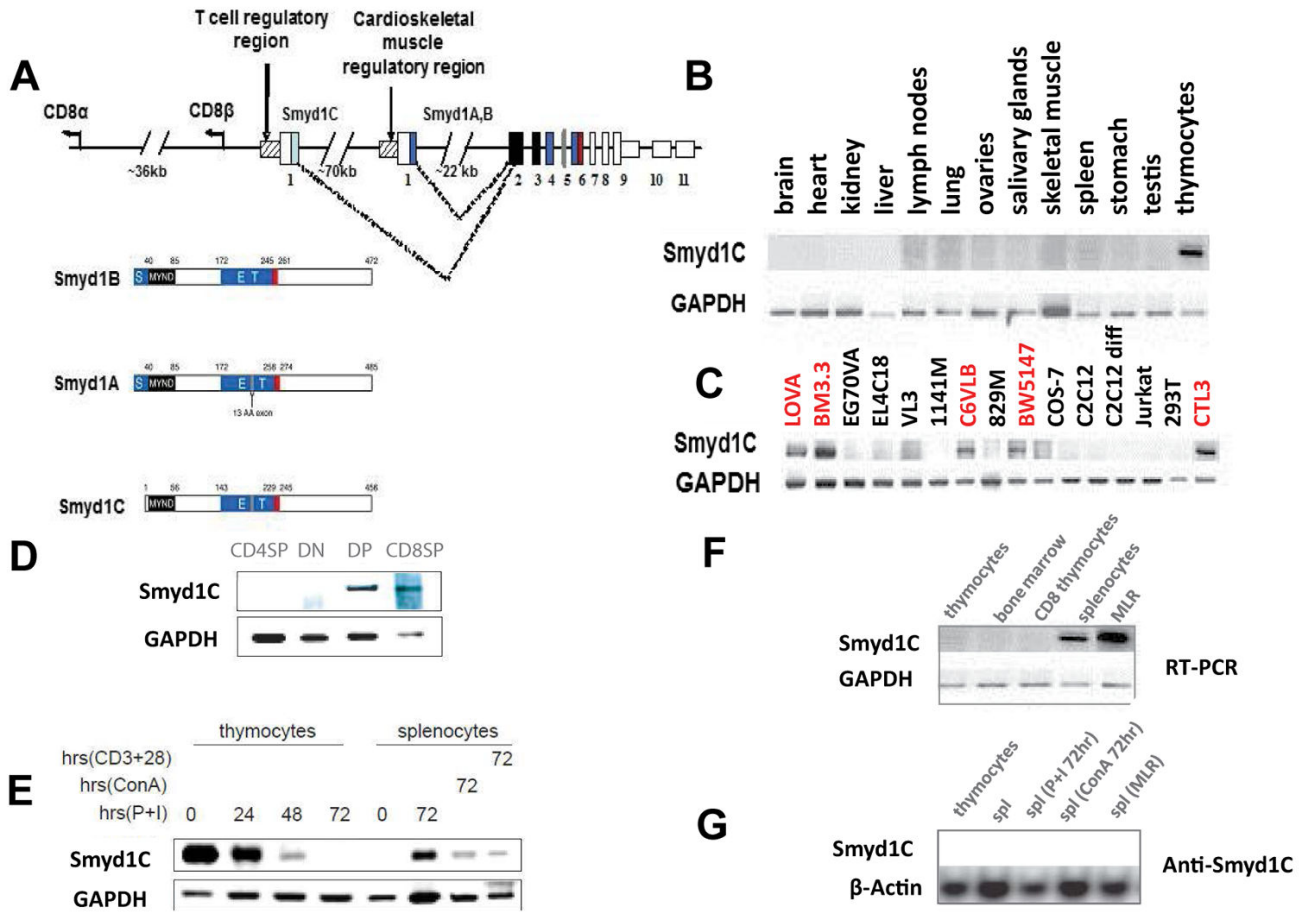
Structure and expression *of Smyd1C* **A.** Schematic of Smyd1 locus and isoforms. *Smyd1C* is transcription is initiated from a start site ~160 bp upsteam of CD8β. The SET domain is split into S and ET portions by the *Mynd* domain. Exons 2–11 are common with 3′ UT (smaller white boxes). The unique exon 1 of *Smyd1C*, light blue; muscle and heart unique exon 1, dark blue; *Smyd1A* unique exon 5, gray; *Smyd1b* unique exon 6, orange. **B.** *Smyd1C* is expressed strongly in mouse thymocytes and weakly in spleen and lymph nodes*. Smyd1C*-specific primers were used to amplify *Smyd1C* cDNA here and in other RT-PCR figures (Table 1) with GAPDH serving as an internal loading control. **C.** *Smyd1C* is expressed exclusively in CD8+ T cell lines. Derivation and references for these cell lines is provided in Materials and Methods. CD8 SP or CD8CD4 DP lines are denoted in red. **D.** *Smyd1C* is expressed in CD8 SP and CD4CD8 DP thymocytes. cDNA was prepared from magnetically isolated CD4SP, CD8SP, DP and DN C57BL/6 thymocytes and subjected to RT-PCR. **E**. Expression of *Smyd1C* is downregulated in response to treatment with CD3 + CD28, Con A or PMA + Ionomycin (P+I). Red cell-deleted, whole thymocytes and splenocytes were cultured with the above stimuli. Cells from each of these conditions were harvested at the hourly time points (indicated only for P+I) and mRNA of *Smyd1C* was examined by RT-PCR. Data shown are representative of a minimum of 3 independent experiments. **F.** *Smyd1C* is expressed most highly in splenocytes following splenocytes, following 6 days of mixed lymphocyte reaction (MLR) using C57BL/6 splenocytes as effectors and irradiated BALB/c splenocytes as targets (details provided in Materials and Methods). **G.** Confirmation of *Smyd1C* expression in splenocytes following 6 days stimulation with P+I or MLR by anti-*Smyd1C* western blotting (faint upper band apparent in some lanes is nonspecific).

*Smyd1C* is a member of the *Smyd* family of proteins which are defined by the presence of a SET domain that is split into two segments by a *MYND* domain. The *Smyd1* gene encodes three distinct isoforms (Figure 1A) [12,13]. *Smyd1A* and *Smyd1B* are expressed exclusively in cardiac and skeletal muscle. They function in cultured cell lines as transcriptional repressors and *in vitro* as H3K4me3-specific histone methyl transferases (HMTases) [14,15]. *Smyd1A/B* null mice die early in embryogenesis from impaired cardiomyocyte differentiation and cardiac chamber morphogenesis [16]. *Smyd1C* transcripts were detected in T cell lines [12], but their function there was not further investigated. Relative *to Smyd1A* and *B, Smyd1C* is encoded by unique upstream promoter region and upstream exon 1, thereby severing the N-terminal half of the SET domain (Figure 1A and re-addressed in Results) [13].

In this study we evaluated the *in vivo* role of *Smyd1C* in T cells. We found that *Smyd1C* accumulates predominantly in the cytoplasm, mitochondria and immunological synapses of activated CD8 cells. *Smyd1C* conditional gene disruption led to impaired clonal expansion of CD8 T cell as a result of heightened levels of apoptosis. *Smyd1C* interacts with FKBP38, Bcl-2, and CaN, but has no HMTase activity toward them or toward conventional histone substrates. Instead*, Smyd1C* is required for dephosphorylation of Bcl-2 and for its efficient targeting to the mitochondrial membrane. Our data identify *Smyd1C* as a critical component of CD8 T cell death via a mechanism uniquely related to ACAD.

### *Smyd1C* is devoid of histone methyl transferase (HMTase) activity and expressed exclusively in CD8+ DP and SP T cells

*Smyd1C* initiates transcription from a poorly consensus Kozak sequence (cccauga) located in the opposite translational orientation just 160bp centromeric to CD8β (Figure 1A). The resulting 31 residue exon 1 shares no significant similarity with any database entries (data not shown). *Smyd1C* exon 1 is spliced in frame to the second exon which is shared with its two orthologues, *Smyd1A* and *B*). This eliminates the S segment of the SET domain (Figure 1A).

Severing the essential N-terminal half of the SET domain predicted that *Smyd1C* would lack HMTase activity. Indeed, that was the case (S-Figure 1A). However, as with its orthologues and paralogues, *Smyd1C* interacted with HDAC1 and displayed transcriptional repression on a synthetic substrate assayed by the Gal4-UAS system (Figures 1B and 1C). While this suggested that a transactivation domain might be retained, *Smyd1C* displayed no global gene expression alteration when over-expressed (data not shown). Thus, we conclude that *Smyd1C* unlikely plays a significant role in transcription.

It was previously reported [12] that *Smyd1C* expression was detected only in CD8+ cell lines and in thymus. Tissue expression survey confirmed that *Smyd1C* was expressed highly in thymus, modestly in spleen and strongly in CD8 T cell lines (Figures 1B and 1C). We further observed that *Smyd1C* transcripts in spleen were induced by Con A and dramatically induced when stimulated under conditions (detailed in Materials and Methods) of a secondary Mixed Lymphocyte Reaction (20 MLR) (Figure 1F, upper panel). *In vitro* 20 MLR mimics the allogeneic response of a recipient haplotype against donor MHC. To further examine the expression of *Smyd1C* in thymocyte subsets, mouse CD4 single-positive (SP), CD8SP, CD4CD8 double-positive (DP) and CD4 and CD8 double-negative (DN) thymocytes were isolated on respective magnetic beads. Levels of *Smyd1C* mRNA were analyzed by semi-quantitative RT-PCR. As predicted by its unique orientation downstream of the CD8 promoter, *Smyd1C* was expressed nearly exclusively in CD8 SP or DP T cells (Figure 1D).

### *Smyd1C* undergoes mitogen-stimulated activation in CD8 T cells

We induced T cell activation by cross-linking the TCR/CD3 complex with immobilized anti-CD3 antibody along with co-stimulatory molecules (eg, anti-CD28) or with 12-phorbol 13-myristate acetate plus ionomycin (P+I). The latter approach bypasses cell-surface signaling by activating protein kinase C and increasing the intracellular Ca^2+^ concentration. We monitored *Smyd1C* transcript expression at 0, 24, 48 and 72 h post-stimulation by RT-PCR. As shown in Figure 1E, we observed upregulation of *Smyd1C* mRNA at 72 h following induction in splenocytes. This was accompanied by a gradual decline in *Smyd1C* transcripts in thymocytes at 24 h and a significant downregulation at 48 h.

It should be noted that all employed modes of stimulation were competent in upregulating *Smyd1C* expression in peripheral lymphocytes, suggesting that *Smyd1C* is an immediate downstream target of activated signaling. Up to 90% of cells in the thymus are DP immature thymocytes, and sustained TCR signaling leads to differentiation of those thymocytes to CD4SP T cells [17]. This is consistent with our observation that *Smyd1C* transcripts in thymocytes were reduced by prolonged TCR stimulation and subsequently expressed primarily in CD8 cells rather than CD4 cells (Figure 1D).

*Smyd1C* is expressed most highly in splenocytes following 6 days of mixed lymphocyte reaction (MLR) using C57BL/6 splenocytes as effectors and irradiated BALB/c splenocytes as targets (Figure 1F; details provided in Materials and Methods). Although the above analyses detected elevated *Smyd1* transcripts, Western blotting allowed direct confirmation of *Smyd1C* protein expression, particularly following activation by P+I, ConA and MLR in splenic T cells (Figure 1G).

### *Smyd1C* partitions within mitochondria and peripheral supramolecular activation clusters of activated CD8 T cells

**Figure 2:**
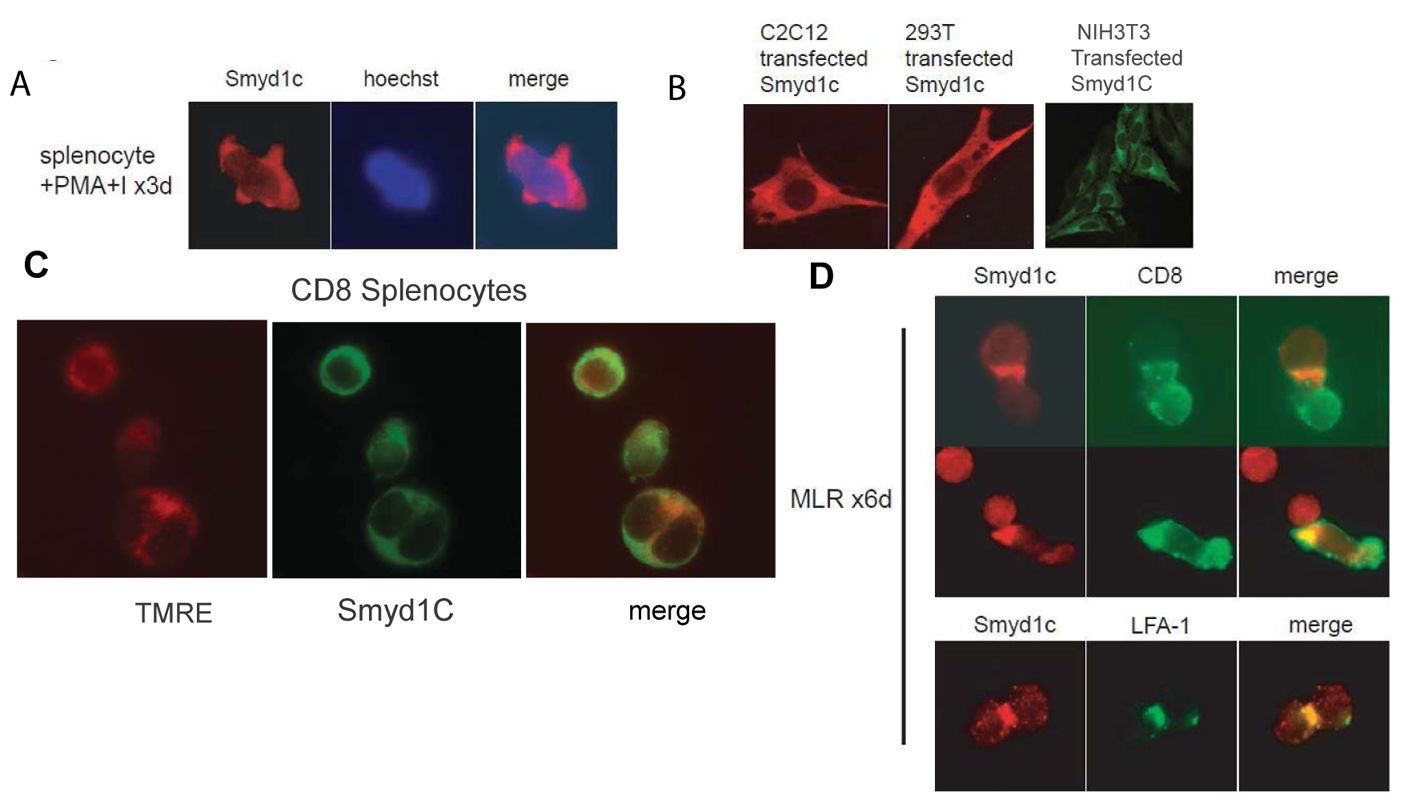
*Smyd1C* partitions within mitochondria and peripheral supramolecular activation clusters of activated CD8 T cells **A.** *Smyd1C* localizes exclusively within the cytoplasm of splenocytes following 6 days P+I stimulation. Nuclei are marked by Hoechst staining. Images here and in subsequent figures were acquired using a 12 bit CCD camera (Model DVC-1312M, DVC, Austin, TX) on a Nikon Diaphot 200 fluorescence microscope. **B.** *Smyd1C* localizes to the cytoplasm following transient transfection into non-lymphoid cells, including C2C12 (immortalized myoblasts), 293 T (transformed human embryonic kidney cells) and NIH3T3 (immortalized fibroblasts). **C.** *Smyd1C* localizes to mitochondria following P+I stimulation of magnetic-bead purified CD8 splenocytes. Green, anti-*Smyd1C*; red, the mitochondrial-specific dye, TMRE. **D**. *Smyd1C* colocalizes with CD8 at the immunological synapse following 6 days of MLR employing C57BL/6 splenocytes as effectors and irradiated BALB/c splenocytes as targets. Upper panel: *Smyd1C* co-localizes with CD8 at the interface of stimulated CD8 T cells and APC. Lower panel: *Smyd1C* localizes at CD8-stimulator-cell peripheral supramolecular activation clusters (pSMACs) as indicated by co-staining with the pSMAC component, LFA-1. Images shown are representative of a minimum of 20 cell pairs.

Immunofluorescence microscopy initially indicated that *Smyd1C* is expressed exclusively within the cytoplasm of splenocytes stimulated by P+I (Figure 2A). Transfection of *Smyd1C* into nonlymphoid cell lines recapitulated this result, indicating that cytoplasmic localization is not T cell-restricted (Figure 2B). On closer inspection, we observed that, following P+I stimulation, *Smyd1C* localized in a punctate pattern, suggesting that at least a sub-fraction may reside in mitochondria. Mitochondrial localization was confirmed by staining with TMRE, a compound that loads specifically into polarized mitochondria (Figure 2C; readdressed below) [18].

Since allogeneic stimulation of peripheral T cells strongly induced *Smyd1C* expression (Figure 1D), we examined *Smyd1C* localization following 6 days of MLR, employing 4–6 wk old C57BL/6 splenocytes as effectors and irradiated BALB/c splenocytes as targets. Slides were fixed and initially stained for detection of *Smyd1C* and CD8. As shown in Figure 2D, a significant fraction of the cells formed stable T cell/APC conjugates. Similar results were obtained when CTL3, a CD8 T cell line, was employed as stimulator (S-Figure 2). Such conjugates are characteristic of supramolecular activation clusters (SMACs) typically observed at CTL–APC interfaces. Indeed, LFA-1, an integrin that marks the peripheral (pSMAC) cluster strongly colocalized with *Smyd1C* at the conjugate interface (Figure 2F).

Previous studies showed that during T cell activation, mitochondria mobilize towards the vicinity of the immune synapse [19–21]. Re-inspection of the images of (Figure 2C) indicated that a large fraction of *Smyd1C* and mitochondria (indicated by TMRE fluorescence) colocalized at cell-cell contacts. Taken together, our data suggest that *Smyd1C* is a critical component of mitochondrial-pSMAC alignment and argue for potential non-nuclear signaling function for *Smyd1C* during the early stages of T cell activation.

#### T cell-specific conditional deletion of Smyd1C

**Figure 3:**
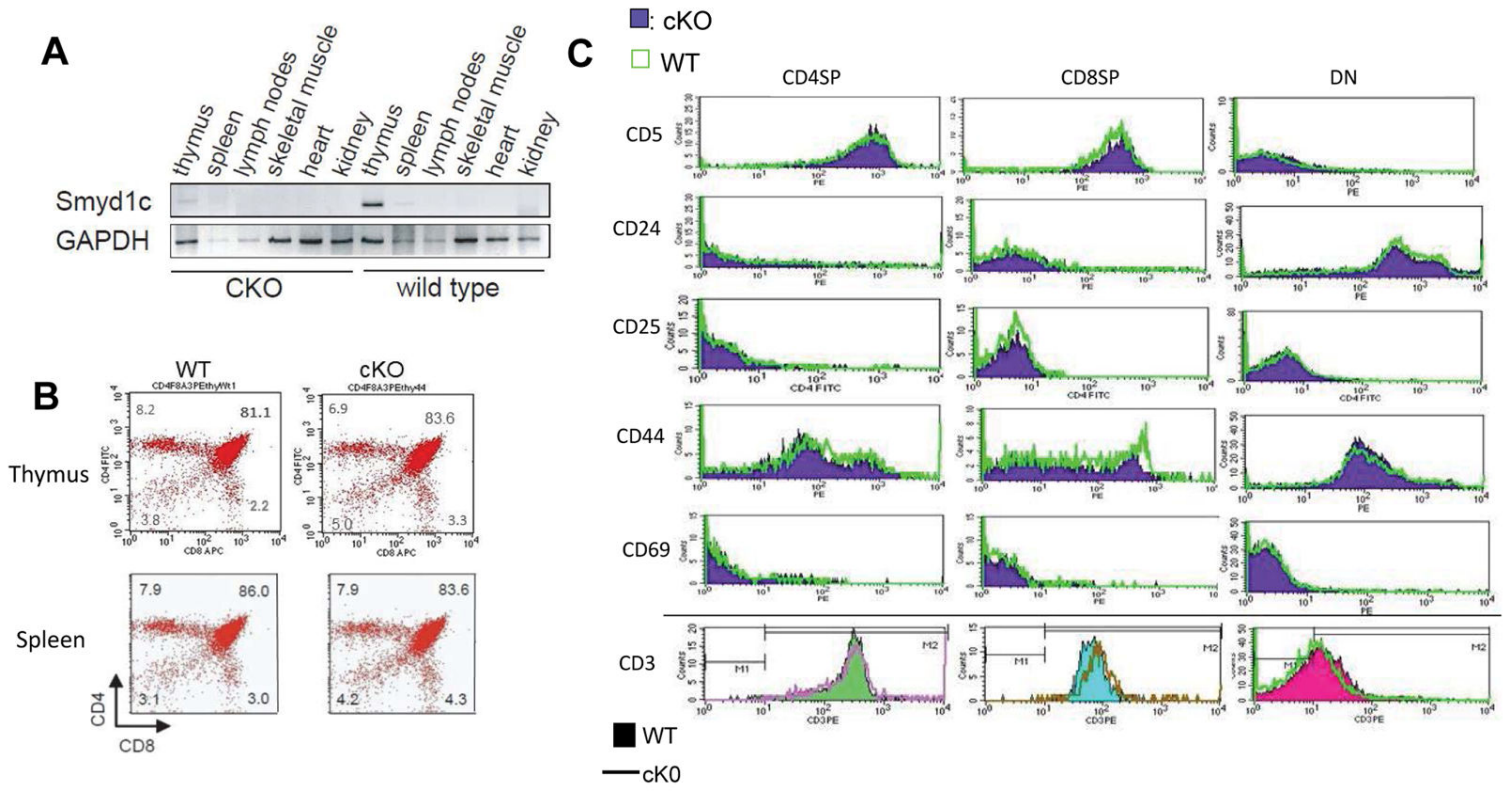
*Smyd1C* Conditional Knockout (CKO) mice show reduction in thymus and show no differences in T cell subsets nor T cell developmental markers **A.** A representative example of *Lck-Cre*-mediated deletion in the thymus and spleen. Total RNA from various tissues was extracted from representative wild-type (WT) and CKO (*Smyd1C^Flox/Flox^;Lck-Cre*) C57/BL6 mice. Semi-quantitative RT-PCR was employed for detecting mRNA levels, and GAPDH served as loading control. KO *Smyd1C* expression is reduced ~80% in thymus and spleen (albeit, WT splenic levels are considerably lower). Expression of paralogues *Smyd1A* and B exclusively in skeletal muscle and heart are unaffected by *Smyd1C* CKO. **B.** FACS profiles show no difference between *Smyd1C* CKO and WT in CD4CD8 DP nor CD4 or CD8 SP subsets in either thymus (upper panel) or spleen (lower panel). **C.** FACS analyses of markers representative of T cell developmental in thymocytes show no change in CKO relative to WT. Upper panels: CKO, red; WT, green; CKO, purple. Lower panel: Equivalent levels of CD3 expression on WT and CKO, indicated for WT as fill (CD4S, green; CD8SP, blue; DN, red) and for CKO as lines (CD4SP, purple; CD8SP, brown; DN, green). Means were established from representative data of 8 mouse pairs.

Germline deletion of *Smyd1* results in embryonic lethality at E9.5, owing to cardiac defects [16]. Transcripts of the two major isoforms, *Smyd1A* and *Smyd1B*, are undetectable in thymus and spleen (S-Figure 3D) [13,16]. This allowed us to analyze the sole function of *Smyd1C* function within the T cell lineage. We employed a conditional *cre/lox* approach (detailed in Materials and Methods and in S-Figure 3) in which a floxed *Smyd1C* allele created in C57BL.6 was crossed into **B6.Cg-Tg *Lck-cre* 548 Jxm/J** driver mice. This system was previously shown to delete CD4 SP and CD4+CD8+ DP cells efficiently, and CD8+ SP cells robustly [22,23].

To confirm *Lck-Cre* specificity in our context, total RNA and genomic DNA from unfractionated thymocytes and other tissues, were examined by semi-quantitative RT-PCR or by end point-PCR using primers that distinguish *WT, floxed, Cre,* and *deleted* alleles. Figure 3A and S-3D show independent and representative examples of *Lck-Cre*-mediated deletion in the thymus and spleen. We estimated that *Smyd1C* expression in thymus and spleen were reduced ~80%, although WT expression levels in spleen are quite low. Adult *Smyd1C* conditional knockout (CKO) mice were indistinguishable from wild type (WT) littermates or from littermates bearing *Lck*, *Cre* or *floxed Smyd1C* alleles alone in viability and overall phenotype (data not shown).

### Resting T cell levels and developmental markers are unaffected by *Smyd1C* loss

Given the highly temporal and T cell-restricted expression pattern of *Smyd1C*, our initial investigation focused on developing thymocytes. In the thymus, immature T cells develop by progressing through distinct stages that are characterized by the expression of different sets of regulatory cell-surface proteins. Mature CD4 and CD8 SP subsets home to peripheral lymphoid tissues [24]. Total thymocytes from *Smyd1C* WT and CKO mice were compared for expression of CD4CD8 DP and SP subsets as well as for T cell development/activation markers, including CD25, CD44, CD3, CD5, CD69 and CD24. FACS profiles indicated that neither T cell subsets nor development markers were significantly altered in splenocytes or in thymocytes of CKO mice relative to WT controls (Figures 3B and 3C; S-). This indicated that *Smyd1C* is not required for T cell development.

### Stimulated *Smyd1C*-deficient CD8 T cells are activation deficient

**Figure 4:**
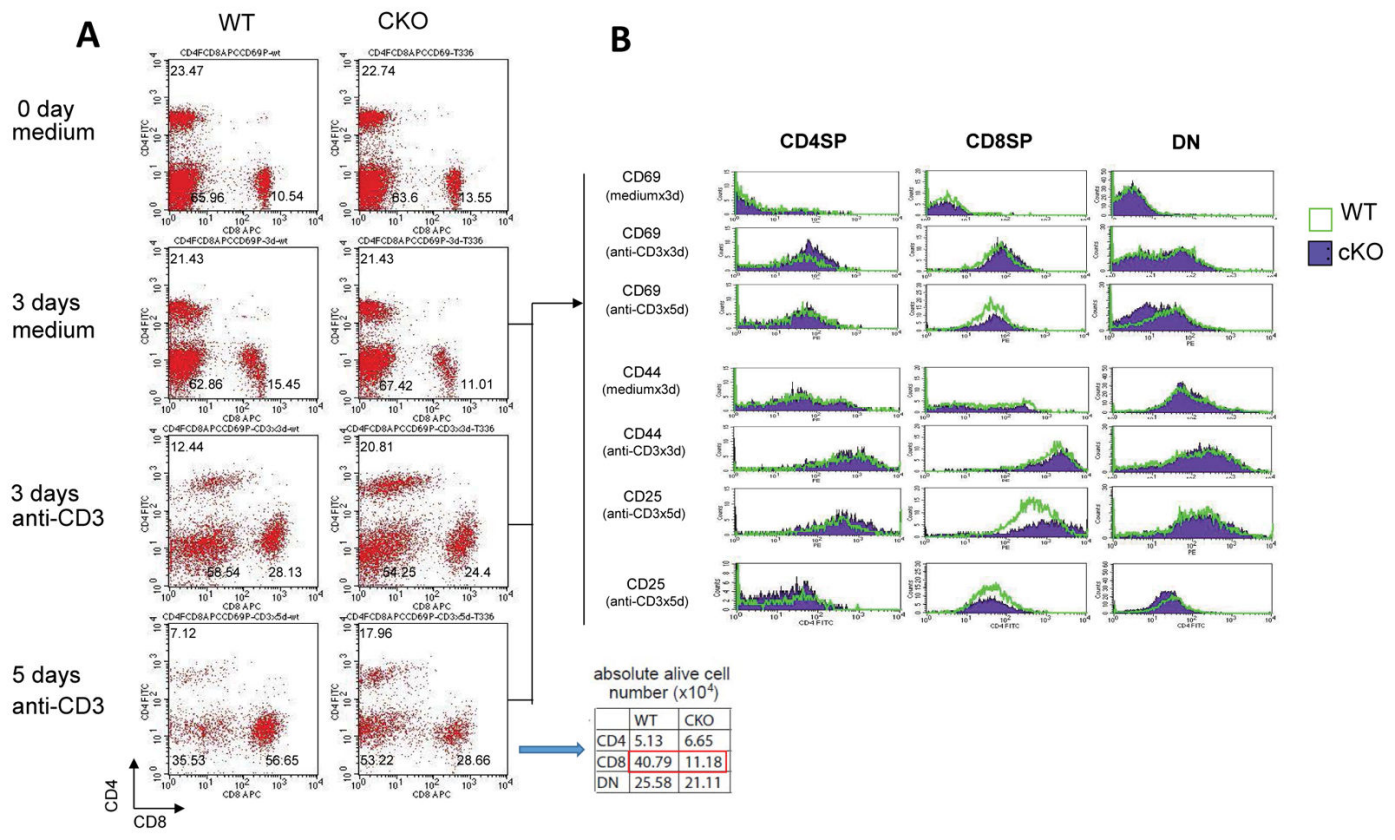
Activation results in reduction in *Smyd1C*-deficient CD8+ splenocytes yet T cell activation markers are unchanged **A.** Activation-induced CD8 SP T cell loss. CD8+ splenic T cells from WT and CKO mice were incubated with plate-coated anti-CD3 (5 ug/ml) and CD28 (5 ug/ml). At the indicated time points, cells were harvested and stained with anti-CD4 (FITC) and anti-CD8 (PE) antibodies. The numbers in each dot-plot represents the percentage of each cell population. The inset provides the absolute cell numbers from day 5 cultures**. B.** Analysis of T cell activation markers in *Smyd1C* CKO spleens. Histograms showing CD69, CD44 and CD25 expression on gated CD4SP, CD8SP and non-T cells from day 5 cultured cells (panel A) were analyzed by FACS. WT, green; CKO, purple. CD25 and CD69 were up-regulated in gated CKO CD8 cells. Plots are representative data of 10 mouse pairs.

**Figure 5:**
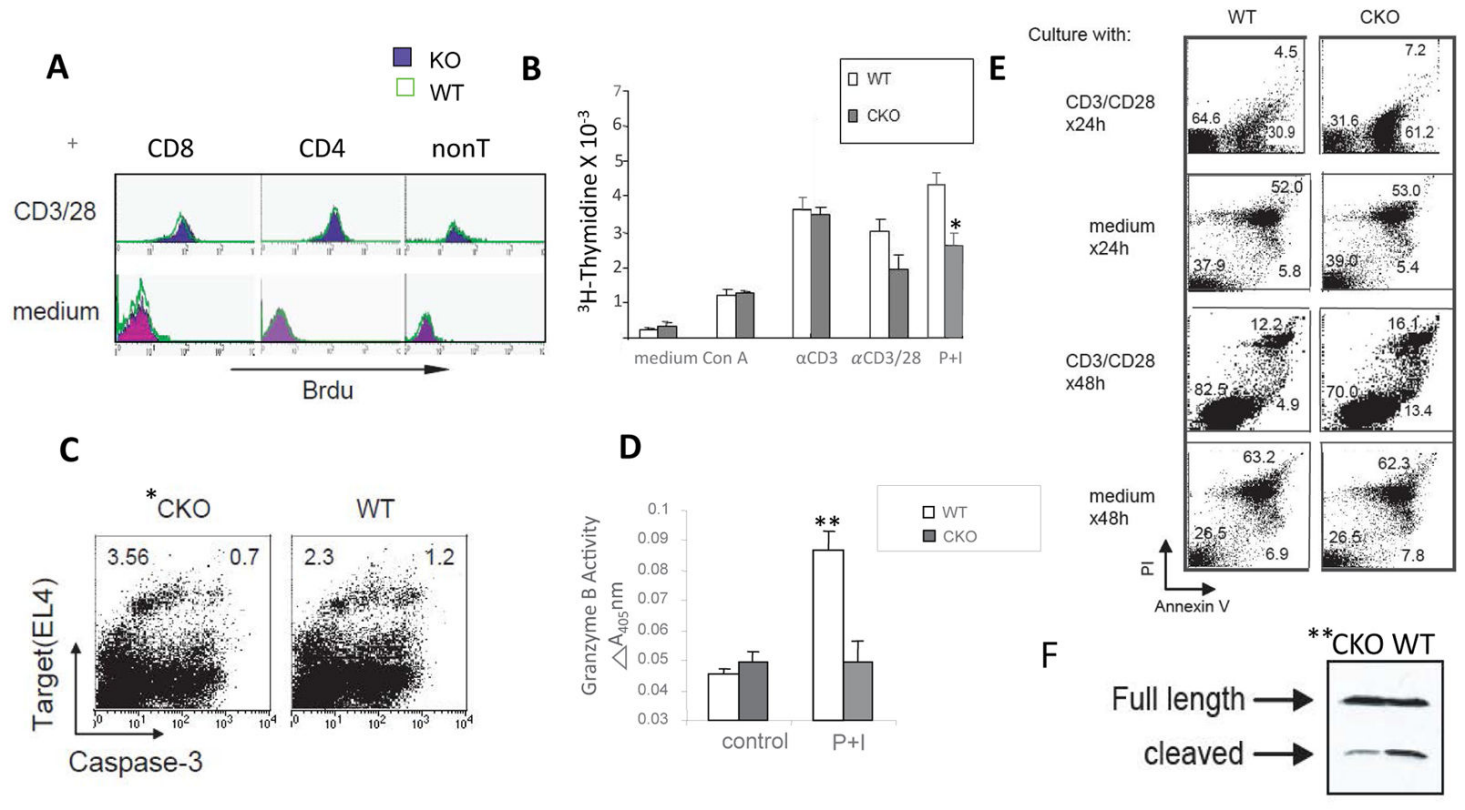
*Smyd1C*-deficient splenocytes undergo activation-induced apoptosis and CTL killing **A.** CKO splenocytes proliferate normally as measured by BrdU incorporation. CD3+ CD28-stimulated WT (green) and CKO (purple) splenocytes were tested for proliferation following intracellular incorporation over 12 h with BrdU (10 uM). CD4 and CD8 cells were then isolated and stained with respective antibodies for FACS analyses. The Y axis is plotted as Mean Fluorescence Intensity (MFI); WT, green; CKO, purple. **B**. CKO thymocyte proliferation is slightly reduced in response to PMA + Ionomycin (P+I) stimulation, Cells were stimulated with ConA, anti (α)-CD3, α-CD3 + α-CD8 or P+I for 3 days and then analyzing for ^3^H-thymidine incorporation as described in Materials and Methods. *p<0.07. **C.** *Smyd1C* CKO cells are deficient in activation-induced apoptosis. CD8 splenocytes were isolated from WT and CKO mice 8 d after infection of mice with LCMV (detailed in Methods and Materials). EL-4 cells were labeled and pulsed with the LCMV peptide NP_396–404_ for 1h and then incubated with splenocytes at an EL-4 to splenocyte ration of 25:1) for 2 h. After 30 min incubation with a cell-permeable, fluorogenic caspase substrate, cells were analyzed by flow cytometry. Shown is a representative plot of 3 experimental replicates (p<0.05). **D.** Release of BLT esterase following stimulation with P+I for 4 h is impaired in CKO splenocytes. Error bars show averages of 3 representative experiments; **p<0.01. **E.** *Smyd1C*-deficient splenocytes actively undergo apoptosis in response to TCR stimulation. Magnetic sorted CD8 cells from WT and CKO mice were incubated with anti-CD3 and anti-CD28 or medium alone. Cells were stained with annexin V and propidium iodide (PI) at the indicated time points and analyzed by FACS. Data shown are representative of 3 independent experiments. **F**. Activated *Smyd1C*-deficient CD8+ splenocytes are defective in killing. Cells fractionated in panel A were harvested at 48 h of culture, and whole cell lysates of WT and CKO CD8+ splenocytes were subjected to Western blotting with anti-caspase 3; upper band represents the full length (uncleaved) and the lower band, the cleaved caspase 3 product. All data are shown as means of 4 independent experiments; **p<0.01.

**Figure 6:**
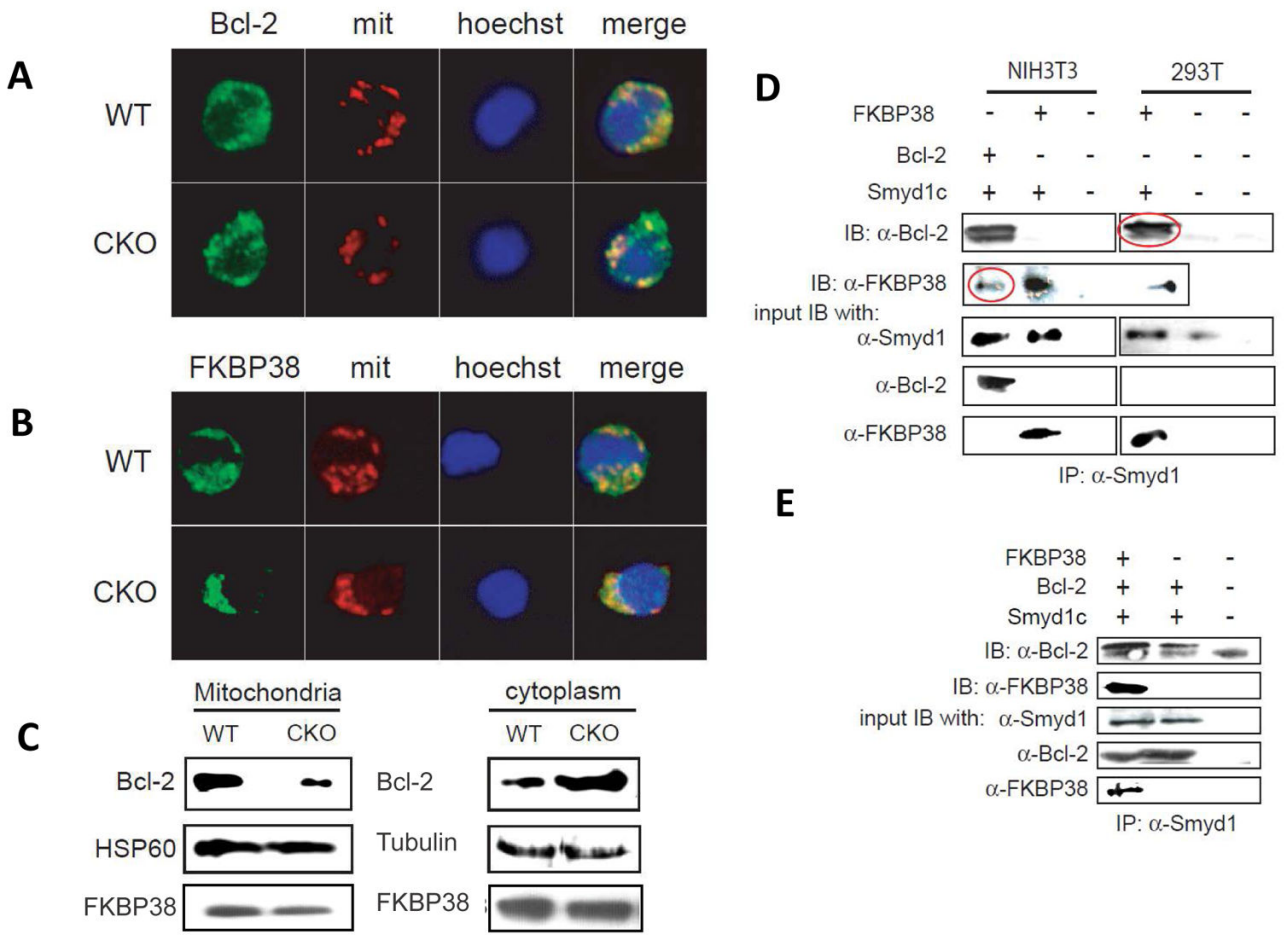
Association of *Smyd1C* with FKBP38 and Bcl1–2 enhances mitochondrial localization **A, B.** Reduced mitochondrial residence of Bcl-2 and FKBP38 in *Smyd1C*-deficient CD8 T cells. Isolated CD8 splenocytes were stimulated with PMA + Ionomycin (P+I) or anti-CD3+anti-CD28 for 24–48 h. Mitochondria were then co-stained with MitoTracker CMXRos (red), anti-Bcl-2 or anti-FKBP38 (green) and Hoechst (blue). Fluorescent images and overlaps were obtained on Leica SP2 AOBS or a Nikon Diaphot 200 fluorescence microscopes. Shown are representative images of >100 captured for each panel. Images shown in A and B are representative of a minimum of 20 cell pairs **C.** Loss of *Smyd1C* leads to significant reduction of Bcl-1 and modest reduction of FKBP3 within mitochondria of CD8 splenocytes. Mitochondria and cytoplasm, isolated as described in Materials and Methods, were lysed and then subjected to Western blotting with anti-Bcl2 and anti-FKBP36. Anti-HSP90 and anti-Tubulin served as loading controls for mitochondria and cytoplasm, respectively. **C.** Interaction of FKBP38 or Bcl-2 with *Smyd1C* in mammalian cells. NIH3T3 or 293T cells were transiently cotransfected with pBK-CMV-*Smyd1C* together with either FLAG-FKBP38 or FLAG-Bcl-2. Whole cell lysates (WCL) were immunoprecipitated with anti-*Smyd1C* and fractionated by SDS-PAGE prior to Western blotting with anti-FLAG mAb. Red circle bands denote immunoprecipitation of corresponding endogenous proteins. WCL representing 5% of the total protein purified served as input for each immunoprecipitation assay; GAPDH served as a loading control. **D.** *Smyd1C* forms a ternary complex with Bcl-2 and FKBP38. NIH3T3 cells were transiently cotransfected with pBK-CMV-*Smyd1C* and FLAG-Bcl-2 with (+) or without (−) FLAG-FKBP38. **E.** WCL was immunoprecipitated, fractionated on 12% SDS-PAGE gels, and then immunoblotted with anti-Bcl-2 or anti-FKBP38. Lanes in which designated antibodies were not employed are indicated by (−). Whole cell lysates (WCL) representing 5% of the total protein purified served as input for each immunoprecipitation assay; GAPDH served as a loading control.

Based on its restricted expression in CD8 T cells and its ability to undergo up-regulation in response to stimulatory signals, we reasoned that *Smyd1C* may function preferentially in activated T cells [12]. Total splenocytes, excluding erythrocytes, derived from *Smyd1C* WT and CKO mice were incubated with P+I or anti-CD3+anti-CD28 to activate T cells. Cells were harvested on days 3 and 5, post-stimulation, and stained with antibodies against CD4 and CD8 as well as against T cell activation markers (CD25, CD44 and CD69). We observed that both percentages and absolute numbers of CD8+ CKO splenocytes and thymocytes were markedly reduced (Figure 4A and S-Figure 5). We also observed downregulation of CD69 and CD25 on gated CKO CD8 T cells (Figure 4B). CD69, a C-type lectin of unknown ligand specificity, is a very early activation marker upregulated on all leukocytes following Ag encounter [25,26]. CD25, a component of the trimeric interleukin 2 receptor (IL-2R), is transiently expressed by CD4+ and CD8+ T cells following TCR activation [27] CD25 appears does not participate directly in ligand binding, but, instead, functions to increase the ligand affinity of IL-2R 10–100-fold [27]. *Smyd1C*-mediated reduction of both CD69 and CD25 is mechanistically linked in that activation of CD69 has been shown to induce CD25 upregulation on CD8+ T cells [28]. However, *Smyd1C* loss did not impair the ability of stimulated splenic T cells to synthesize IL-2 and INF-γ as determined by intracellular staining and RT-PCR (S-Figure 6 and data not shown).

Thus, we reasoned that an underlying contributor to the above phenotype might be failure of activated *Smyd1C*-deficient CD8 splenocytes to proliferate. To evaluate, magnetic bead-isolated CD8 splenocytes from *Smyd1C* CKO and WT mice were stimulated with anti-CD3+anti-CD28 and subjected to 12hr intracellular incorporation of BrdU into newly synthesized DNA. FACS analysis detected no significant deficiency in *Smyd1C* CKO incorporation into CD8 or CD4 T cells (Figure 5A). However, in an alternative approach, in which we measured cell proliferation by ^3^H-thymidine incorporation via several conditions, detected a modest reduction (statistically, a trend towards significance; p ≤ 0.07) in proliferation of P+I stimulated CKO CD8 splenocytes (Figure 5B). Different outcomes using these two types of proliferation analyses have been previously observed [29]. Collectively, we conclude that activated *Smyd1C*-deficient CD8 T cells proliferate at near normal levels.

### *Smyd1C*-deficient CD8 T cells are impaired in killing and in target cell elimination

To assess whether *Smyd1C* deficiency perturbs target-cell elimination mediated by cytotoxic T lymphocytes (CTLs), mice were infected with the murine Lymphocytic Choriomeningitis Virus (LCMV; detailed in Materials and Methods). LCMV elicits a vigorous CTL response against a defined array of MHC class I–restricted viral epitopes [30]. Target EL4 cells were labeled with a fluorescent probe and pulsed with nucleoprotein epitope NP_396–404_. These were then co-incubated with fresh splenocytes obtained from CKO and control mice 8 days following LCMV infection in the presence of a fluorogenic caspase substrate to allow detection of substrate cleavage by FACS. As shown in Figure 5C, the number of target cells undergoing caspase cleavage in *Smyd1C* CKO mice was moderately but significantly reduced relative to WT.

CTL-mediated killing is mechanistically related to granular release by CTL during recognition. Consistent with the above reduction in caspase cleavage, the activity of BLT esterase, a substrate used as a measure of granzyme release, was significantly reduced in CKO mice relative to WT controls (Figure 5D). However, the mRNA levels of two main granular components, Granzyme B and perforin showed no significant reduction (S-Figure 6).

These data indicated that *Smyd1* CKO mice suffer a principle defect in CD8-mediated killing, albeit conventional factors that mediate granular release are unscathed.

#### Smyd1C-deficient CD8 T cells undergo activation-induced apoptosis

Activation of Caspase 3, the “executioner” of cell death, through proteolytic cleavage of its inactive zymogen into activated p17, plays a central role in apoptosis. Therefore, we monitored cleavage of caspase-3 in response to TCR signaling. Using isolated CD8 T cell whole cell lysates stimulated for 3 days with P+I, immunoblotting with anti-Caspase 3 mAb revealed that cleavage of CKO CD8 T cells was strongly reduced (Figure 5F).

These data suggested that *Smyd1C* inhibits activated CD8 T cells from initiating apoptosis and explains, at least in a major part, why CD8 cell numbers were severely reduced by *Smyd1C* loss (Figure 4A) yet proliferative indices were, at best, only modestly impaired (Figures 5A and 5B).

### *Smyd1C* employs neither classical AICD nor ACAD to restrict CD8 T cell responses

As the immune response wanes, activated lymphocytes are removed in two ways: Those re-stimulated near the end of the immune response die by activation-induced cell death (AICD), whereas those activated, but not re-stimulated, die by activated cell autonomous death (ACAD) [1–4]. Several features noted above suggest that *Smyd1C* does not promote AICD. These include failure to activate IL-2, IFNγ and CD96/FAS—quintessential markers of AICD (S-Figure 6) [1–4]. Further, CTLA-4, which elicits a potent block of FasL expression and AICD [30] was unaffected by *Smyd1C* CKO (S-Figure 6). These results were consistent with previous observations that AICD does not require TCR re-stimulation and is independent of death receptor engagement [2].

ACAD is typically regulated by the NF-kb family as well as by the intrinsic cell death pathway involving members of the Bcl-2 family [1,2,4,31,32]. However, mRNA levels of both pro- (e.g., Bim, Bid, Bcl-^XL^) and anti- (e.g., Bcl2, Bcl_XL_ and Mcl-1) apoptotic Bcl-2 family members as well as their effortors (caspases 3 and 8) were unaffected by *Smyd1C* CKO (S-Figure 6).

These results argue against *Smyd1C* as a regulator of either AICD-or ACAD-dependent CD8 Tcell responses. Its action is independent of IL-2, IFNγ and death receptor signaling, and the expression of to pro or anti-apoptotic Bcl-2 family members is not disrupted.

### Association of *Smyd1C* with the immunophilin FKBP38 enhances mitochondrial localization of Bcl-2

**Figure 7:**
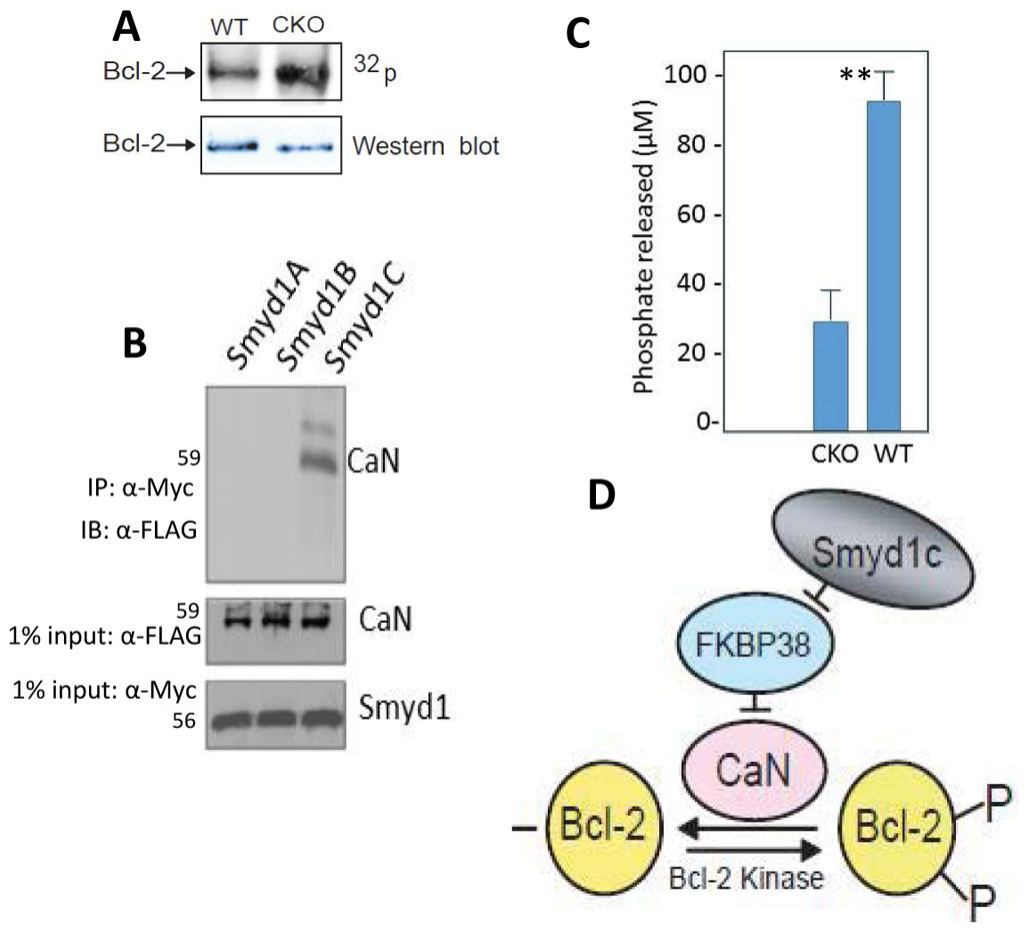
*Smyd1C* enhances phophorylation of Bcl-2 via interaction wtih and activation of Calmodulin **A.** Loss of *Smyd1C* results in reduction of phosphorylation of Bcl-2. PMA + Ionomycin-activated splenic CD8 T cells were labelled with [^32^P]orthophosphoric acid, immunoprecipitated (IP) with polyclonal anti-Bcl-2, and fractionated by SDS/PAGE. Following transfer to nylon, half of the filter was exposed to autoradiography (16hr at −80°C), while the other half was subjected to immunoblotting with a Bcl-2 monoclonal Ab. Shown are representative data of 3 independent experiments. **B.** *Smyd1C*, but not *Smyd1A* or *Smyd1B* interacts with Calcineurin (CaN). NIH3T3 cells were co-transfected with FLAG-CaN and either pBK-CMV-*Smyd1A*, -B or -C. Two days later, WCLs were fractionated by SDS-Page and subjected to Western blotting subsequently probed with anti-CaN or a pan-anti-Smyd1 polyclonal Ab. WCL representing 5% of the total protein purified served as input for each immunoprecipitation assay; GAPDH served as a loading control. Shown are representative data of 3 independent experiments. **C.** *Smyd1C* is essential for maximal CaN phosphatase activity. WT and *Smyd1C* CKO CD8+ splenocytes were purified over magnetic beads. WCLs were prepared and then employed for colorimetric determination of CaN phosphatase activity using a phosphopeptide as substrate (detailed in Materials and Methods). Detection of free phosphate release was measured by formation of a complex between malachite green molybdate and free orthophosphate that absorbs at 620 nm. Data shown are the mean concentration of phosphate released measured in four independent experiments; **p<0.001. **D.** A schematic model by which activated T cell fate is determined by *Smyd1C*. The model postulates that upon CD8 T cell stimulation, activated CaN forms a complex with Bcl-2. Meanwhile, *Smyd1C* is gradually up-regulated and may exert a dominant negative effect by sequestering FKBP38. This results in de-repression of CaN by FKBP38 and induction of dephosphorylation and mitochondrial localization of Bcl-2, resulting in anti-apoptotic action of Bcl-2.

To search for an alternative mechanism by which *Smyd1C* protects against CD8 death, we performed yeast two-hybrid screening using *Smyd1* as a bait to screen a mouse T cell cDNA library (Materials and Methods). Among the positive candidates, we identified FK506-Binding Protein (FKBP38; S-Figure 7. FKBP38), an immunosuppressant of FK-506 Binding Protein, serves an important function in mitochondria-mediated apoptosis by regulating anti-apoptotic Bcl-2 [5,33,34] This prompted us to examine if the expression of FKBP38 and Bcl-2 were affected by loss of *Smyd1C*. This was not the case, as analysis of P+I activated splenocytes revealed similar levels of FKBP38 and Bcl-2 protein and transcripts in *Smyd1C* CKO and WT mice (Figure 6 and data not shown).

Binding of FKBP38 to Bcl-2 has been shown to induce mitochondrial localization of their complex [5,33]. Thus, we reasoned that *Smyd1C* might regulate apoptosis by altering the intracellular localization of FKBP38, Bcl-2 or both. Under physiological conditions, both FKBP38 and Bcl-2 localize primarily within mitochondria, although smaller quantities are found in ER, Golgi and the nuclear envelope. Double IF staining of P+I activated splenic T cells revealed that the mitochondrial localization of Bcl-2 was more prominent in *Smyd1C* WT than in CKO cells (Figure 6A). We observed no significant difference for FKBP38 localization (Figure 6B). To confirm these results, we isolated [28]; mitochondria from activated peripheral T cells. As shown in Figure 6C, mitochondrial Bcl-2 was significantly reduced, while cytoplasmic Bcl-2 was significantly increased in *Smyd1C* CKO cells. Localization of FKBP38 again showed only modest alteration (Figure 6C). These observations suggested that *Smyd1C* promotes mitochondrial localization of Bcl-2.

### *Smyd1C* associates with Bcl-2 and FKBP38

Next we examined whether Bcl-2 associates with *Smyd1C*. Following co-transfection of *Smyd1C* and Bcl-2 into NIH3T3 cells, both transfected and endogenous (red circled) Bcl-2 were detected in anti-Smyd1 immunoprecipitates (IPs; Figure 6D, left panels). Reciprocally, anti-*Smyd1C* IPs also contained both transfected and endogenous (red circled) FKBP38 (Figure 6D, right panels). Finally, following triple-transfection of *Smyd1C*, FKBP38, and Bcl-2 into NIH3T3 cells, each were IP’d by *Smyd1C* (Figure 6E). The *Smyd1C* doublets observed in Figures 6D and 6E likely represent phosphorylated and unphosphylated forms (readdressed below).

These results raised the possibility that Smyd1, Bcl-2, and FKBP38 reside in the same complex. They further suggest that *Smyd1C* regulates Bcl-2 localization by associating directly, or indirectly with it and Bcl-2. As Bcl-2 anti-apoptosis activity is dependent upon its mitochondrial localization, such association would provide a mechanism by which *Smyd1C* regulates apoptosis in activated CD8 cells (readdressed below).

### *Smyd1C* enhances phophorylation of Bcl-2 while associating with Calmodulin

Proteins are often regulated by post-translational modifications that control their activity and conformation. In most instances, phosphorylation of Bcl-2 has been associated with its inactivation and restriction of its localization predominantly to the ER [30–32]. This led us to hypothesize that reduced mitochondrial occupancy of Bcl-2 in *Smyd1C*-deficient mice may result from alteration in its phosphorylation state. To address this, P+I activated peripheral T cells were labeled with [^32^P]-orthophosphoric acid and then IP’d using a polyclonal Bcl-2 antibody. The IPs were split into two fractions, and each was fractionated by SDS/PAGE. Half was exposed to autoradiography (16hr at −80°C), while the other half was subjected to immunoblotting with a Bcl-2 mAb. As shown in Figure 7A, the level of phosphorylated Bcl-2 was significantly increased in *Smyd1C* CKO T cells.

Association of Bcl-2 with the calcium-calmodulin-dependent protein phosphatase Calcineurin/PP2B phosphatase (CaN) was shown to prevent TCR-mediated apoptosis by blocking intracellular calcium signaling [35,36]. CaN was further shown to bind directly to both Bcl-2 and FKBP38 [33,37–39]. As shown in Figure 7B, CaN also immunoprecipitated specifically with *Smyd1C*, but not *Smyd1A* or *B*, in CD8+ splenocytes. We find it noteworthy in this regard that a measurable loss in CaN-mediated phosphatase activity was observed in lysates prepared from stimulated CKO CD8 splenocytes (Figure 7C).

### Hypothetical mechanism by which activated T cell fate is regulated by *Smyd1C*

Our data indicate that reduction of *Smyd1C* triggers an earlier onset of activated T cell death and results in an abbreviated immune response. In Figure 7D, we illustrate an unconventional ACAD model for the underlying mechanism. We suggest that *Smyd1C* acts as a scaffold for Bcl-2, FKBP38 and CaN. This hypothetical complex insures Bcl-2 phosphorylation-based mitochondrial association and anti-apoptosis of activated CD8 T cells. In turn, loss/down-regulation of *Smyd1C* expression leads to dissociation of the complex, CaN-mediated dephosphorylation of Bcl-2 and consequently, activation-induced apoptotic cell death. This model is readdressed in the Discussion.

## Discussion

TCR stimulation of primary T cells generates a pool of active effector cells through a process of activation and expansion, followed by cell death [37,38,40]. It is important to regulate the onset of cell death to maintain a balance between the effector phase and subsequent cell death. An abbreviated effector phase could result in an inadequate immune response, whereas a prolonged effector phase could lead to the accumulation of overly activated cells. The mechanism by which this balance is controlled is not well understood. While it is known that Fas signaling contributes to the death of some activated T cells, it certainly does not control the death of all, or even most, as indicated by several reports that have shown that activated T cells display little variation in their rate of death in the absence of Fas signaling [3,41,42]. Experiments by others strongly suggest that many activated T cells die through an intrinsic cell death pathway (Activated Cell Autonomous Death; ACAD), ACAD often is executed via Bcl-2 family members and mitochondrial release of cytochrome C [41–45]. The death of the majority of activated T cells responding to foreign antigen *in vivo* can be prevented by over-expression of Bcl-2 [42,45]. Bcl-2 inhibits ACAD by *associating* with Bim at the mitochondrial membrane to block its apoptotic function and/or to alter permeabilization of the outer mitochondrial membrane [33,46]. The molecular mechanism(s) that control this intrinsic cell death pathway are unclear.

In contrast to its muscle-specific, enzymatically active isoforms, *Smyd1C* is exclusively expressed in CD8 SP thymocytes, spenocytes and some CTL cell lines (Figures 1C–1G) [12,13]. Expression of *Smyd1C* is robustly induced by TCR stimulation (Figures 1D–1G). While there are no obvious phenotypic alterations found in *Smyd1C*-deficient resting T cells, their CD8 cells respond to TCR stimulation with a high rate of cell death (Figures 4A, 5C and 5E). *Smyd1C* lacks the essential N-SET domain and thus, demonstrates no HMTase activity (S-Figure 1). Thus, we searched for a non-enzymatic mechanism. We found that *Smyd1C* acts via a new twist on the classical Activated Cell Autonomous Death (ACAD).

Bcl-2 resides on the cytoplasmic face of the mitochondrial outer membrane, endoplasmic reticulum (ER), and nuclear envelope [6,47]. The mitochondrial localization of Bcl-2 is essential for its anti-apoptotic function [6,8]. Evidence has shown that FKBP38 regulates apoptosis through interacting with Bcl-2 and anchoring it on the mitochondrial membrane [48,49]. Our results show that *Smyd1C* interacts with endogenous FKBP38 and Bcl-2 in co-immunoprecipitation experiments from cultured mammalian cells (Figures 6D and 6E). We further demonstrate that the mitochondrial localization of FKBP28 is normal, whereas that of Bcl-2 is significantly reduced in activated CD8 T cells of *Smyd1C* CKO mice (Figures 6A–6C). This suggested that *Smyd1C* inhibits apoptosis by targeting Bcl-2 to the mitochondria.

*In vivo*, Bcl-2 exists in varying states of phosphorylation and these states influence its subcellular location, its binding to pro-apoptotic family members, and ultimately, its anti-apoptotic activity [50–52]. Phosphorylated Bcl-2 is known to be predominantly localized within the ER where it is prevented from binding to the BH3-only pro-apoptotic protein Bim [50,53]. Bcl-2 is also known to prevent TCR-mediated apoptosis by blocking intracellular calcium signaling [54]. This results from its association with Calcineurin (CaN), a calcium-calmodulin-dependent protein phosphatase that was found to bind directly to both Bcl-2 and FKBP38 [6,37–39]. FKBP38 is an inherent inhibitor of CaN and was found to promote protein dephosphorylation [27]. In addition, activated CaN results in neutralization of the anti-apoptotic action of Bcl-2 [6,37–39]. We found that CaN interacts with *Smyd1C*, and its phosphatase activity is impaired following *Smyd1C* CKO or Smyd1B knockdown in C2C12 myocytes (Figures 7B and 7C; data not shown). As anticipated from CaN enzymatic loss, phosphorylated Bcl-2 levels were significantly elevated in activated CD8 splenocytes (Figure 7A).

Taken together, these findings suggest that Bcl-2 may be regulated by *Smyd1C* through its association with FKBP38 and calcineurin. Perhaps these three proteins function in a ternary complex, although such stoichiometry remains to be formally established.

We propose in Figure 7D the following model: In resting T cells, which express modest levels of *Smyd1C*, the balance between survival and death of T cells is maintained by a complex set of ACAD and AICD mechanisms. However, in stimulated CD8 T cells, an increase of intracellular calcium ions activates CaN which, in turn, forms a complex with Bcl-2 (or potentially a Bcl-2-*Smyd1C* complex). As the level of *Smyd1C* is gradually up-regulated upon stimulation, it may exert a dominant negative effect by interacting with, and thereby sequestering, FKBP38. This results in de-repression of CaN by FKBP38. De-repressed CaN then can induce dephosphorylation of Bcl-2, potentially enhanced binding to Bim, and mitochondrial localization of Bcl-2. Thus, apoptosis is prevented, and CD8 T cells are maintained in an adequate effector phase.

FKBP38 binds to mTOR and inhibits its anti-apoptosis activity [55,56]. Thus, anti-apoptosis achieved via *Smyd1C* through a potential FKBP38-mTOR pathway cannot be excluded and should be investigated in the future. Further studies are required to determine how *Smyd1C* controls the phosphorylation state of Bcl-2 and to better define the role of *Smyd1C* at the immunological synapse. Finally, we suggest that extension of these studies may lead to better understanding of how activated T cells escape apoptosis in autoimmune disorders or in T cell lymphomas [1,47].

## Methods

Mice protocols/approvals and description of T cell lines [24,34,57–68] Bai et al. [17] and Takayama et al., [18] are provided in Appendix 1. Details of our conditional T cell knockout (CKO) of *Smyd1C* employing B6.Cg-TgLck-cre548Jxm/J driver mice [22,23] and genotyping approaches [17] for screening are detailed in Appendix 1. Standard implementation of suspension cultures, mixed lymphocyte reaction (MLR) [12,34] and flow cytometry [12,63] were previously described and detailed in Appendix I. Immunoprecipitation and western blotting details were described previously [14,15]. Mitochondria were isolated and stained by the method of [28]. CTL assays were as described previously [14,15,68]. BLT-esterase activity was measured in supernatants as described by Takayama et al. [66]*. In vivo* metabolic labeling and IP were previously described [14,15]. The Matchmaker Gold Yeast Two-Hybrid 63system was used to screen for *Smyd1C* binding partners. Calcineurin Phosphatase activity was determined by a colorimetric kit (Abcam#13946).

## Supplementary Material

Suppl Table

Suppl files
