# Supplementary figures and images for: Smyd1C Mediates CD8 T Cell Death via Regulation of Bcl2-Mediated Restriction of outer Mitochondrial Membrane Integrity
Source: J Cell Signal (Los Angel). Author manuscript; Available in PMC 2017 Nov 22. (PMC5699232; doi:10.4172/2576-1471.1000163)

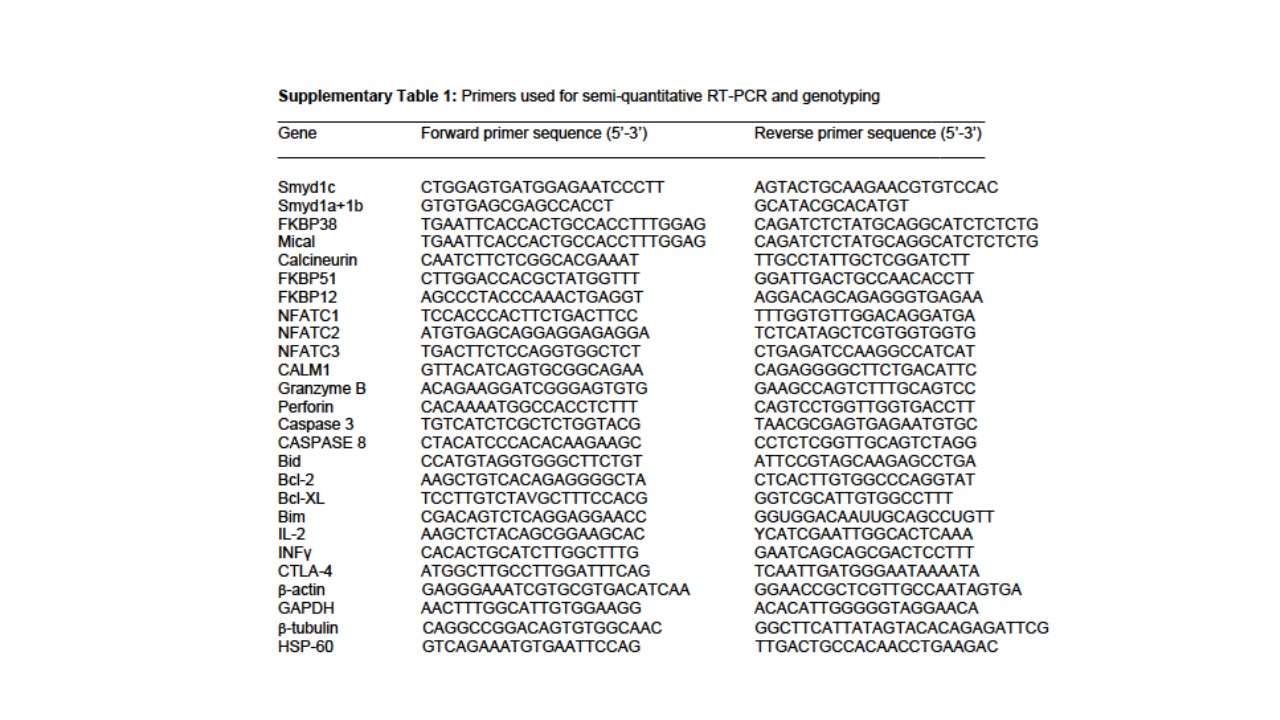

Supplement: Suppl Table [file NIHMS915246-supplement-Suppl_Table.JPG]
