# Supplementary material for: Smyd1C Mediates CD8 T Cell Death via Regulation of Bcl2-Mediated Restriction of outer Mitochondrial Membrane Integrity
Source: J Cell Signal (Los Angel). Author manuscript; Available in PMC 2017 Nov 22. (PMC5699232; doi:10.4172/2576-1471.1000163)
Supplement: Suppl files [file NIHMS915246-supplement-Suppl_files.docx]

**Supplemental Methods**

**Mice**: All mice used in this study were 6-12 week old C57/Bl6. They were maintained in specific pathogen-free conditions according to institutional guidelines. The mouse protocol was approved by the Institutional Animal Care and Use Committee at the University of Texas at Austin (Austin, TX).

**T cell lines:** We employed the following T cell lines/clones for isolation of RNA and subsequent survey of Smyd1C expression: Lova, a C57/BL6 splenic OT1 transgenic CD8 T cell specific for Listeria OVA21;

BM3.3, an alloreactive CD8 T cell clone expressing the BM3.3 TCR22;T cell clone derived from the EL-4 thymoma23; VLA3, CD4+CD8+ DP T cell clone24; 111M, small cell carcinoma25 C6VLB, CD8 murine lymphoma 829M26, MNU induced, CD4 lymphoma27 ; BW5127, DN T cell lymphoma28.

**Conditional knoCKOut (CKO) of Smyd1C in T cells**: A targeting vector was engineered with LoxP sites flanking the common exons 2 and 3 of Smyd1 isoforms followed by a NeoR cassette and a third LoxP site. This was electroporated into SM1/129S6 ES cells and selected with G418 and gancyclovir. Clones were screened for homologous recombination by Southern analysis after digestion with BglII and SalI or with BglII and KpnI and hybridization with probes 2 and 3, respectively. Correctly targeted ES clones were injected into C57BL/6 blastocysts to create chimeric mice, which were mated with C57BL/6 females to create germline knock-in mice. These mice were crossed to mice carrying the ubiquitous deleter E2A-Cre transgenic strain, and their offspring were screened for the NeoR deletion by Southern analysis. We crossed Smyd1flox/flox mice lacking E2A-Cre with mice expressing the Cre recombinase under the control of the distal promoter of the mouse Lck gene (B6.Cg-Tg Lck-cre 548Jxm/J; Jackson Laboratory).

**Genotyping:** The method for genotyping the conventional deletion of Smyd1 was described previously17. The floxed Smyd1 and Lck-Cre alleles were detected by PCR. Primer sequences are provided in Table For RT-PCR, total RNA of cultured cells or tissues was isolated with TRI REAGENT (MRC, Inc. Cincinnati, OH). Total RNA was reversed transcribed into cDNA by using the poly (dT) oligonucleotide (SUPERSCRIPT II, GIBCO–BRL).

**Cell isolation and in vitro suspension cultures:** Thymocyte subsets were magnetically separated by sequential positive selection of anti-CD4 and anti-CD8 microbeads using MACS MultiSort Kits according to the manufacturer’s instructions (Miltenyi Biotec Inc. Auburn, CA). CD4 and CD8 splenocytes were separated by anti-CD4 and anti-CD8 microbeads, respectively. Cells (5x105/ml) were cultured for the indicated times with a combination of phorbal-12-myristate-13-acetat (PMA) (10ng/ml) and Ionomycin (0.5ug/ml) (Sigma), or anti-CD3 (5 ug/ml)/CD28 (5ug/ml) (eBioscience, San Diego, CA) or Concanavalin A (ConA; Sigma). To measure Brdu incorporation, 10 uM Brdu (Sigma) was added to cultures 12 hours before harvesting.

**Mixed Lymphocyte Reaction (MLR):** Secondary MLRs were performed as previously described16,29. A C57BL/6-Lyt2aLyt3a (M16)29 (H-2b) X BALB/c (H-2d) secondary mixed leukocyte culture was prepared essentially as described previously17. Briefly, spleen cells (1.2 X 106/ml) prepared from 6- to 8-wk old MI6 mice were incubated with irradiated (30 Cy) BALB/c spleen cells (1.2 X 106/ml) for 12 to 14 days. Viable cells (2 X 105/ml) were then re-stimulated with irradiated BALB/c spleen cells (1.5 X 106/ ml). Cells were collected 72 h later and used for RNA preparation.

**Flow cytometric analyses:** Where indicated, cells were harvested and stained with fluorochrome-conjugated antibodies as previously described30. Monoclonal antibodies (BD Pharmingen) were directed at the following cell surface molecules: TCR (H57-597); CD4 (RM4-5), CD8α (53-6.7), CD25 (7D4), CD5 (53-7.3), and CD69 (H1.2F3). Intracellular staining of IL-2 (JES6-5H4), INF- (XMG1.2) and BrdU (B44) was performed following the manufacturer’s instructions. Fluorescence was measured with a FACSCalibur flow cytometer and analyzed with CELLQuest software (Becton Dickinson).

**Flow-cytometry CTL (FCC) assay:** FCC assays were performed as previously described31. Briefly, C57BL/6 (H2b) mouse splenocytes were isolated 8 days after infection with LCMV Armstrong strain (provided by R. Ahmed) as effector cells. TFL4 dye labeled EL4 cells were pulsed with peptides NP396−404 (FQPQNGQFI) synthesized at the UT core facility, Austin, TX as target cells. Effector and target cells were incubated at a ratio of 25:1 for 2 hours, prior to addition of a cleavable fluorogenic caspase 8 substrate inhibitor for detection by FACS (Abcam Staining Kit ab65614).

**Constructs and transient transfection**. The mammalian expression vectors pBK-CMV-Smyd1A, Smyd1B, Smyd1C and pFLAG-CMV4 were described previously32. Briefly. pcDNA3-Flag-FKBP38, -Flag-Mical and -Flag Helicase expression vectors were supplied by Dr. Deepak Srivastava (Southwestern Medical Center, Dallas, Texas). The sequences of all constructs were confirmed by DNA sequencing. FuGENE6 reagent (Boehringer Mannheim) was used to transiently transfect 293T cells according to the instructions of the manufacturer. 80-90% confluent cells were harvested and plated at a density of 2-2.5 × 106 cells per 100-mm plate 24 h prior to transfection. 4-8 µg of total DNA was used per 100-mm plate. Cells were harvested 48 h after transfection.

**Immunoprecipitation and western blotting:** Whole cell lysates were incubated with a hamster anti-mouse Smyd1 monoclonal antibody generated against the common C-terminal half of all Smyd1 isoforms32, or with anti-FLAG mAb M2 (Sigma) or with caspase-3 (Cell Signaling Technology, Inc. Danvers, MA) for 1 h at 4 °C, followed by incubation for 1 h with protein-A sepharose beads (Sigma). After extensive washing with RIPA buffer, the precipitated proteins were analyzed by SDS−PAGE and transferred to nitrocellulose. Membranes were probed with the Smyd1 monoclonal antibody or FLAG M2 and developed with the enhanced chemiluminescence analysis coverslips. Calcineurin was detected with a rabbit polyclonal anti-calcineurin antibody (1:1000 dilution; Affinity Research Products, Exeter, UK).

**Immunostaining:** The method used for immunostaining was previously described33. Briefly, cells settled on coverslips were treated with 1 mg/ml poly-L-lysine, for 20–30 min prior to fixation with 4% paraformaldehyde. To co-stain with mitochondria, MitoTracker Red CMXRosred (Molecular Probes) was added to live cells for 5 minutes before cells were seeded. Fixed cells were permeabilized with ice-cold methanol/acetone (1:1) for 20 minutes. After washing twice, samples were blocked with a solution of 5% goat serum and 0.1% Tween-20 in PBS for I hr and then additionally washed three times. Samples were incubated with primary antibody and secondary antibody sequentially for 1 hr, with washing following each incubation. Coverslips were then mounted on slides using ProLong Gold antifade reagent (Invitrogen, CA). The primary antibodies used are anti-FKBP38 (a gift from Dr. Frank Edlich, Germany), anti-BcL-2(Becton Dickinson, CA), anti-LFA-1(Becton Dickinson, CA) and anti-Smyd1. The secondary antibodies were obtained from Santa Cruz Bio. Inc. Either a Leica SP2 AOBS confocal microscope or a Nikon Diaphot 200 fluorescence microscope using a 12 bit CCD camera (Model DVC-1312M, DVC, Austin, TX) were used for fluorescence imaging.

**Lymphocytic Choriomeningitis Virus (LCMV) Cytotoxic T lymphocyte (CTL) assay:** This procedure has been described in detail elsewhere34 Briefly, Five 6- to 8-week-old female CKO and WT C57B6-H2b mice were immunized intraperitoneally with one injection (0.05 ml; 200,000 p.f.u) of LCMV Armstrong (clone 13 strain). Virus-infected mice were sacrificed at day 8 post-infection, and splenocytes (defined as effectors”) were collected. Single cell suspensions were prepared from each of the spleens. After a 6-h incubation at 37"C, the cells were pelleted, supernatants were collected, and targets were produced in vitro (under BL/2 level containment) by infecting EL-4 T cells (MHC-I H2b) with LCMV at Multiplicity of Infection (MOI)=2-4) for 48 h at 37C. Resulting CTLs were isolated in vitro by passage of the splenocytes over CD8-magnetic beads. Flow cytometry-based CTL assays were performed by addition of a fluorogenic Caspase-3 substrate (Ac-DEVD-AMC; BD Biosciences Cat. No. 556449).

**Mitochondrial isolation and staining:** Mitochondria were isolated from cultured CD8 T cells by the method of Chappell and Hansford (1972) in medium comprising 0.25 M sucrose, 5 mM Tris–HCl and 2 mM EGTA (pH 7.4 at 4°C) (STE buffer). Crude mitochondria were purified according to Pagliarini et al (2008). Briefly, 0.5 ml of crude mitochondria (about 30–40 mg ml−1) was layered on top of a stepwise density gradient of 2 ml 80%, 6 ml 52% and 6 ml 26% Percoll in a 50‐ml centrifuge tube. The gradient was centrifuged at 41,100 g for 45 min, and mitochondria (collected from the 26–52% interface) were diluted in STE buffer and centrifuged at 12,000 g at 4ᵒC for 10 min. Mitochondria were washed with an additional 2 ml of STE buffer, re-centrifuged and the resulting pellet was resuspended in a small volume of STE buffer for functional experiments. Mitochondria were identified by staining with TMRE (tetramethylrhodamine ethyl ester; Abcam113852) according to the manufacturer’s instructions. TMRE is a cell permeant, positively-charged, red-orange dye that readily accumulates within active mitochondria due to their relative negative charge.

**In vivo metabolic labeling and immunoprecipitations:** In vivo metabolic labeling and immunoprecipitations were done as previously described35. Immunoprecipitations were carried out by adding polyclonal anti-Bcl-2 antibody (Santa Cruz Bio. Inc.) to [32P]-orthophosphoric acid- (Amersham Corporation)-labeled cell lysates. The samples were immunoprecipitated and divided into 2 equal aliquots. One was used for Western blotting with monoclonal anti-Bcl-2 antibody; the second was separated by 10% SDS gel and vacuum dried prior to autoradiography.

Na-benzyloxycarbonyl-l-lysine thiobenzyl ester (BLT) esterase release assay. BLT-esterase activity was measured in supernatants using the methods described by Takayama et al.36.

**Yeast two‐hybrid screening**: The Matchmaker Gold Yeast Two‐Hybrid system was used to isolate Smyd1‐interacting partners according to the protocol described in the Yeastmaker Yeast Transformation System 2 User Manual (PT1172-1, Cat No. 630439 to screen a human T lymphocyte cDNA library (Clontech). Briefly Smyd-1 was cloned into pGBKT7 and then was transformed and integrated into the yeast strain Y2H. The ensuing bait-integrated yeast strain was subjected to expression test and checked for autoactivation and toxicity (as determined by color and growth status of the diluent yeast) following spreading transformants on a series of nutrient-deficient, selective agar plates. Co-transformation of the library cDNA (prey) and the purified pGBKT7-Smyd1 (bait) into Y187 yeast strain allowed interaction between prey and bait. Yeast colonies grown on selective agar medium (Double dropout media containing 40 µg/ml X-a-Gal and 200 ng/ml Aureobasidin A) were then harvested and subjected to further analysis.

**Calcineurin Phosphatase Activity Assay:** We employed a colorimetric kit (Abcam #139461) for non-radioactive colorimetric determination of Calcineurin (CaN) phosphatase activity as described by the vendor. Briefly, using the RII phosphopeptide as substrate for CaN, detection of free phosphate release was measured by formation of a complex between malachite green molybdate and free orthophosphate that absorbs at 620-640 nm.

**SUPPLEMENTAL FIGURE LEGENDS**

**S-Table 1**. Primers used for semiquantitative RT-PCR and genotyping.

**S-Figure 1:** Smyd1C mediates transcriptional repression on model substrates but does not possess histone methyltransferase activity. A. GAL4-Smyd1C represses VP16-mediated transcription. 10T1/2 cells were transfected with GAL4-DBD, GAL4-Smyd1C or GAL4-Smyd1A in the presence or absence of the LexA-VP16 activator (0.2 ug) along with pL8G5-luc (0.3 ug). pRL-TK (0.5 ug) was cotransfected for internal standardization.1X represents 0.3 ug of GAL4-Smyd1C DNA. The DNA amount was held constant by adding GAL4-DBD. Percent activity was determined in relation to GAL4-DBD alone. Three independent experiments were performed to calculate the mean and standard error. B. Smyd1C has no Histone Methyl Transferase (HMTase) activity. In vitro HMTase previously shown to detect activity for Smyd1A and Smyd1B against trimethyl-H3K3 was assessed for purified GST-fusions of Smyd3, Smyd1A and Smyd1C (100ng) plus 20mM S-adenyl-methionine by western blotting with anti-dimethyl-H3K4 (upper panel) or a negative control, anti-dimethyl-H3K9 (middle panel). Lower panel: Smyd1A, but not Smyd1C, shows HMTase activity against histone octamers. Recombinant, purified wildtype (WT, H3K4m3) and mutant (H3K4/3-m3) histones were purified individually following expression in E coli, mixed, and dialyzed to form octamers as previously described70. Octamers were purified by gel filtration, and HMTase assays were conducted as in (B). C. Smyd1C shows no methyltransferase activity toward E34. Smyd1A, Smyd1B or Smyd1C was immunoprecipitated (IP’d) from transiently transfected 293T cells and used in in vitro MTase assays on GST-E34, a subcloned 34kD region of the skeletal muscle-specific transactivator, skNAC. The MTase reaction products were separated by SDS-PAGE, stained with Commassie blue, and subjected to fluorography (top lanes). Western blot analysis was performed with anti-FLAG mAb; bottom lanes, ~10% of IP’d Smyd1; middle lanes, equal amounts of GST-E34 (middle panel) employed as input.

**Supplementary Figure 2:** Stable T cell/APC conjugate formation at Smyd1C and CD8 interfaces following activation by Mixed Lymphocyte Reaction. C57/BL6 splenocytes were isolated from 4-6 wk old WT C57BL/6 mice; CD8 T cells were isolated from the CD8+ T cell line, CTL3. MLR was performed for 6 days employing splenocytes as effectors and irradiated CTL3 cells as targets (detailed in Materials and Methods). A. Conjugate formation of CD8+ effectors and targets. MLR cultures were stained with anti-Smyd1 mAb and imaged and captured by Phase contrast as detailed in Materials and Methods. The small, round cells in CTL3 panels are the stimulators. Images are magnified 60-100-fold. B. Double immunofluorescence staining of Smyd1C+CD8+ conjugates. C57BL/6 splenocytes were incubated with irradiated stimulator cells (2000 rad; BALB/C) and 10% ConA supernatant for 5 days. Cells were incubated as described in Materials and Methods, fixed and stained with anti-Smyd1 and anti-CD8 Abs. Images are magnified 60-fold. A significant fraction of the cells formed stable T cell/stimulator conjugates characteristic of supramolecular activation clusters (SMACs). Data are representative of a minimum of 20 conjugates analyzed.

**Supplementary Figure 3:** Conditional Smyd1c-deficiency in T cells. A. A targeting vector was constructed by introducing 2 Lox P sites (triangles) flanking coding exons 2 and 3 and a third flanking the NeoR. The Smyd1C floxed allele was generated by crossing initially with ubiquitously expressed E2A-Cre. Arrows indicate the location of PCR primers that were used for analysis of Cre/Lox recombination in different tissues. B. Southern analysis of targeted ES cells and PCR genotyping. For the 5’arm, Southern analysis was performed following digestion with BglII and KpnI, and hybridization with probe 2 (pr2) to distinguish a 4.5 kb wild type allele and a 7.5 kb targeted allele. For 3’ arm, genomic DNA was digested with BglII and SalI and then the following blot was hybridized with probe 3(pr3) to distinguish the 13.7kb wild type allele from the 7.0 kb targeted allele. Tail tip genomic DNA was used for PCR to genotype. P1 and P2 primers were used for detecting floxed allele (larger band) and wild type (smaller band); Neo primers and cre primers were used for deleted allele and Lck-cre, respectively (lower panel). C. Various tissue DNAs isolated from a representative Lck-cre/+; Floxed Smyd1c/+ mouse were subjected to PCR amplification using the primer set shown in A. For each set of PCRs, the upper and lower bands represent un-recombined Lox-p sites and wildtype alleles, respectively. Sample tail tip* was from mouse Floxed Smyd1c/+. D. A representative example of Lck-Cre-mediated deletion in the thymus and spleen. Total RNA from various tissues was extracted from representative wild-type (WT) and CKO (Smyd1cFlox/Flox;Lck-Cre) C57/BL6 mice. Semi-quantitative RT-PCR was employed for detecting mRNA levels of Smyd1A, B and C. GAPDH served as loading control. Note that Smyd1A and B are not expressed in hematopoietic tissues. cKO Smyd1C expression is reduced ~80% in thymus and spleen (albeit, WT splenic levels are considerably lower; please see Figure 3A for an independent duplicate).

**Supplemental Figure 4:** Expression of developmental surface markers in Smyd1C CKO thymocytes is normal. Thymocytes were isolated and fractionated for CD4SP, CD8SP, DN and DP subsets on magnetic beads. Cells were then stained with fluorochrome-conjugated antibodies to identify T cell developmental markers (CD3, CD5, CD44, and CD25). No differences, measured in 3 independent analyses, were observed between WT and CKO mice. Data shown are representative of 3 independent experiments.

**Supplemental Figure 5:** Smyd1C-deficient CD8 splenic cells undergo activation-induced CD8 SP T cell loss. CD8+ splenic T cells from WT and CKO mice were incubated with plate-coated anti-CD3 (5ug/ml) and CD28 (5ug/ml). At the indicated time points, cells were harvested and stained with anti-CD4 (FITC) and anti-CD8 (PE) antibodies (detailed in legend to Fig.4). The live cells were counted directly following isolation by Trypan Blue exclusion, and their absolute numbers were plotted as percentages of total cells.

**Supplemental Figure 6**: Gene expression in Smyd1c-deficient splenocytes. Splenocytes from CKO and WT mice were incubated with plate-coated anti-CD3 (5ug/ml) and CD28 (5ug/ml) for 5 days. Total RNA was prepared with Sepasol (Nacalai Tesque, Japan). cDNA was generated with SuperScript reverse transcriptase (Invitrogen) and amplified by PCR. Levels of the indicated transcripts were determined by semi-quantitative RT-PCR. β-Actin levels were normalized as loading controls of input RNAs. Shown are representative data from 4 independent measurements.

**Supplemental Figure 7:** Confirmation of Smyd1C interacting proteins isolated by yeast two-hybrid screening. Yeast two hybrid clones isolated using Smyd1 as bait were screened and isolated as described in Materials and Methods. Candidate interacting proteins were tagged at their N-termini with 4X-FLAG. Each clone was co-transfected with Smyd1C into NIH3T3 cells. Following 48hr incubations, transfected cells were converted to whole cell lysates (WCL) and subjected to immunoprecipitation (IP) with Smyd1C. Co-IPs were fractionated by SDS-PAGE and then subjected to Western blotting with anti-Smyd1C. Clones passing our screen (as detailed in Materials and Methods) were Mical, E34 (A 34kD breakdown product of skNAC), Helz RNA helicases (helicase), and FKBP38. Although unanticipated, we as yet do not know if the former 3 proteins are relevant to the studies reported here. The latter clone, FKBP38, was employed for further analysis.
